# Clinical outcomes of palliative self-expandable metal stent placement in right- and left-sided malignant colon obstruction: A Honam Association for the Study of Intestinal Disease (HASID) multicenter study

**DOI:** 10.1097/MD.0000000000030156

**Published:** 2022-08-26

**Authors:** Hyung-Hoon Oh, Sung-Bum Cho, Ji-Yun Hong, Dong-Hyun Kim, Hee-Chan Yang, Sang-Wook Kim, Jun Lee, Seong-Jung Kim, Yeom-Dong Han, Geom-Seok Seo, Gun-Young Hong, Ho-Dong Kim, Dae-Seong Myung, Hyun-Soo Kim, Young-Eun Joo

**Affiliations:** a Department of Internal Medicine, Chonnam National University Medical School, Gwangju, Republic of Korea; b Department of Internal Medicine, Chonbuk National University Medical School, Jeonju, Republic of Korea; c Department of Internal Medicine, Chosun University College of Medicine, Gwangju, Republic of Korea; d Department of Internal Medicine, Wonkwang University College of Medicine, Iksan, Korea; e Department of Internal Medicine, Kwangju Christian Hospital, Gwangju, Republic of Korea; f Department of Internal Medicine, Saint Carollo Hospital, Suncheon, Republic of Korea.

**Keywords:** colon cancer, obstruction, outcome, self-expandable metal stent

## Abstract

Self-expandable metal stent (SEMS) placement is commonly used for palliation of left-sided malignant colorectal obstruction (MCO). However, right-sided MCO is usually treated surgically. Recent studies that compared palliative SEMS insertion and emergency surgery in right-sided MCOs have reported conflicting results. This study aimed to compare the effectiveness of palliative SEMS placement in left-sided MCOs and right-sided MCOs and to investigate the predictive factors for clinical success and risk factors for complications.

Data from 469 patients who underwent palliative SEMS placement for MCO at 6 hospitals in the Honam province of South Korea between 2009 and 2018 were reviewed. Among them, 69 patients with right-sided MCO and 400 patients with left-sided MCO who underwent SEMS placement for palliative purposes were enrolled. Clinical success, overall survival, complications, and predictive factors for clinical success and risk factors for complications were included as the main outcome measures.

The clinical success rates were 97.1% (65/67) in right-sided MCO patients and 88.2% (353/400) in left-sided MCO patients. Complications including stent migration, tumor ingrowth, outgrowth, perforation, bacteremia/fever, and bleeding occurred in 10.1% (7/69) of right-sided MCO patients and 19.9% (79/400) of left-sided MCO patients. The mean overall survival of right-sided MCO was 28.02 months and 18.23 months for left-sided MCO. In multivariate logistic regression analysis, T3 stage tumors and the use of uncovered stents were significant factors for the clinical success of SEMS. The use of covered stents and performance status score of 0 to 2 were independent significant risk factors for complications.

Palliative SEMS placement in right-sided MCO showed better clinical success rates than left-sided MCO. The use of uncovered stents is recommended for higher clinical success rates and lower complication rates.

## 1. Introduction

Colorectal cancer is the third-most common cancer worldwide, and its incidence in patients younger than 50 years of age has been increasing.^[1]^ Approximately 8% to 29% of patients with colorectal cancer experience malignant colorectal obstruction (MCO) and require emergency decompression.^[2,3]^ Classically, MOC has been treated with emergency surgery with stoma formation or primary resection. However, recent studies have reported that emergency surgery is associated with higher morbidity and mortality compared to elective surgery.^[4,5]^ Therefore, the need for alternative treatment options has emerged. After Dohmoto,^[6]^ first reported a case of self-expandable metal stent (SEMS) insertion for palliation of MCO, numerous studies have evaluated the efficacy of SEMS insertion in MCO.

SEMS insertion can be performed for 1 of 2 purposes: to act as a bridge to curative surgery and for palliation. In the former case, SEMS insertion has been accepted as a treatment option, especially in left-sided MCO, as it shows similar overall survival (OS), lower complication rates, and lower stoma formation rates but higher recurrence rates and perforation risk, compared to emergency surgery.^[7–9]^ In a palliative setting, SEMS insertion is the preferred treatment option for MCO, especially in left-sided MCO.^[7]^ Compared to emergency operation, SEMS insertion was associated with shorter hospital stays, lower intensive care unit care rates, lower stoma formation rates, and earlier chemotherapy initiation with no significant difference in OS and morbidity rates.^[10–13]^ However, some studies have reported that late complications were more common in SEMS insertion than in emergency operation.^[13,14]^ Therefore, knowing factors associated with complications in SEMS insertion may help prevent them and also help clinicians explain their occurrence or its probability to patients.

In right-sided MCO, emergency resection and primary anastomosis have been considered the treatment of choice.^[15]^ The results of recent studies that compared palliative SEMS insertion and emergency surgery in right-sided MCO are quite conflicting. One study reported that the SEMS insertion group experienced lower temporary stoma formation rates and higher SEMS-related complication rates compared to emergency surgery group with no difference in the morbidity and mortality rates.^[16]^ Another study reported lower clinical success and lower patency for SEMS insertion compared to emergency surgery.^[17]^ However, the number of patients who underwent palliative SEMS insertion in right-sided MCO was very small in both studies.

In this retrospective study, we aimed to compare the effectiveness of palliative SEMS placement in left-sided MCO with that in right-sided MCO, and investigate the factors associated with clinical success and complications.

## 2. Materials and Methods

### 2.1. Patient enrollment

Data from 802 patients, each of whom underwent SEMS placement for MCO between January 2009 and December 2018 at 1 of 6 hospitals (4 university hospitals and 2 community hospitals) in the Honam province of South Korea, were collected retrospectively. The hospitals are affiliated with the Honam Association for the Study of Intestinal Diseases. Among these patients, 469 underwent SEMS placement on palliative purposes. We diagnosed MCO using a combination of clinical symptoms and imaging studies, including plain abdominal radiography, computed tomography and colonoscopy. The institutional review board of each hospital approved this study.

### 2.2. Procedure protocol

Each SEMS placement was performed by 1 of 7 gastroenterologists (S.W.K., J.L., G.Y.H., H.D.K., D.S.M., H.S.K., and Y.E.J.). First, we advanced a 2-channel therapeutic endoscope (Olympus, Tokyo, Japan) or colonoscope (Olympus, Tokyo, Japan) to the obstruction site. We then passed an endoscopic retrograde cholangiopancreatography catheter (MTW Endoskopie, Wesel, Germany) through the obstruction using a guidewire (Glidewire; Terumo, Tokyo, Japan). After the removal of the guidewire, we injected contrast dye (Gastrografin; Schering, Berlin, Germany) into the catheter to check the length, morphology, and location of the obstruction. Then, a guidewire was used to guide the SEMS delivery catheter to be placed on the proximal side of the obstruction site. We deployed the stent under endoscopic and fluoroscopic guidance. We estimated the obstruction length under fluoroscopy by measuring the inserted catheter length from each margin of the obstruction. An appropriate length of the stent that spanned at least 2 cm beyond each margin of the obstruction was chosen. Plain abdominal X-rays were taken on the 1st and 2nd days after the procedure to check the position of the SEMS in all patients. Every patient who underwent SEMS placement was assessed for clinical success, and complications.

### 2.3. Definitions and outcomes

The cecum, ascending colon, and transverse colon were regarded as the right-sided colon, and the descending colon, including the span from the splenic flexure to the rectosigmoid colon, was regarded as the left-sided colon. Clinical success was defined as the resolution of symptoms, including abdominal pain, abdominal distension, failure of gas and feces passage, and resolution of bowel distension on plain abdominal radiography, within 48 h after the procedure. The patient’s performance score was calculated based on the Eastern Cooperative Oncology Group scale of performance status (PS).^[18]^ The patient’s clinical TNM stage was diagnosed with imaging studies including computed tomography based on the 8th American Joint Committee on Cancer colon cancer staging.^[19]^ Perforation, migration of stent, tumor ingrowth, tumor outgrowth, bleeding, and bacteremia/fever were included in complications.

### 2.4. Statistical analysis

Continuous variables were expressed as mean ± standard deviation. Categorical variables were expressed as frequencies and percentages. Student *t*-test, chi-square test, or analysis of variance was used as appropriate. Factors associated with clinical success and risk factors for the complications of SEMS placement were assessed using a logistic regression model. Survival probability analysis was performed using the Kaplan–Meier method. Statistical significance was set at *P* < .05. All data were analyzed using Statistical Packages for the Social Sciences, version 27.0 (SPSS Inc., Chicago, IL).

## 3. Results

### 3.1. Baseline characteristics of enrolled patients

A total of 69 patients with right-sided MCO and 400 patients with left-sided MCO were enrolled in this study. Age, sex, PS score, abdominal operation history, length of obstruction, completeness of obstruction, TNM staging, the use of uncovered stent, and stent length were similar between the 2 groups. The tumor was located in the ascending colon (n = 16), hepatic flexure (n = 28), or transverse colon (n = 25) in right-sided MCO; and the splenic flexure (n = 32), descending colon (n = 17), S-D junction (n = 22), sigmoid colon (n = 147), rectosigmoid junction (n = 120), or rectum (n = 62) in left-sided MCO (Table 1).

**Table 1:** Baseline characteristics of enrolled patients.

Variable		Right-sided (n = 69)	Left-sided (n = 400)	*P* value
Age (yr)	Mean ± SD	69.8 ± 14.2	68.3 ± 13.1	.407
Gender	Male	31 (44.9%)	165 (41.3%)	.567
	Female	38 (55.1%)	235 (58.7%)	
BMI (SD), kg/m^2^	Mean ± SD	22.0 ± 3.3	21.6 ± 3.2	.412
PS score	0–2	52 (75.4%)	305 (76.3%)	.873
	3–4	17 (24.6%)	95 (23.8%)	
Abdominal operation history		18 (26.1%)	90 (22.5%)	.513
Tumor location		Ascending 16 (23.2%)	Splenic flexure 32 (8.0%)	
		Hepatic flexure 28 (40.6%)	Descending 17 (4.3%)	
		Transverse 25 (36.2%)	SD junction 22 (5.5%)	
			Sigmoid colon 147 (36.8%)	
			Rectosigmoid junction 120 (30.0%)	
			Rectum 62 (15.5%)	
Length of obstruction (mm)	Mean ± SD	37.6 ± 11.2	37.0 ± 13.1	.562
Complete obstruction		50 (72.5%)	283 (70.6%)	.772
T (n = 103)	T3	8 (47.1%)	57 (66.3%)	.133
	T4	9 (52.9%)	29 (33.7%)	
N (n = 108)	N0	1 (5.6%)	18 (20.0%)	.476
	N1	8 (44.4%)	31 (34.4%)	
	N2	9 (50.0%)	40 (44.4%)	
	N4	0 (0%)	1 (1.1%)	
Metastasis		61 (88.4%)	371 (92.8%)	.216
Uncovered stent		49 (71.0%)	271 (67.8%)	.591
Length of stent		84.9 ± 16.6	86.7 ± 17.3	.410

BMI = body mass index, PS score = performance status score, SD = standard deviation.

### 3.2. Clinical outcomes and complications after palliative SEMS placement

**Figure 1.**
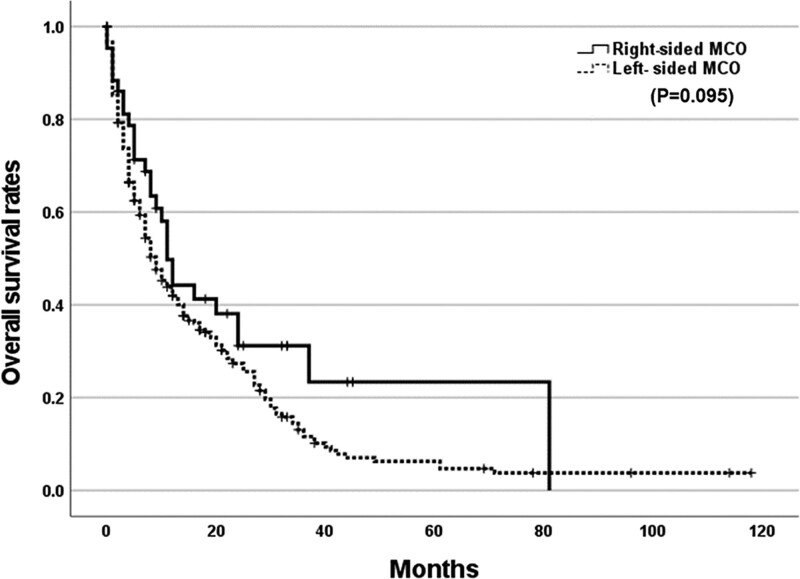
Comparison of overall survival based on location of MCOs. Mean follow-up time for right-sided MCO was 15.31 ± 13.80 mo and left-sided MCO was 18.49 ± 20.10 mo. The mean overall survival of right-sided MCO was 28.02 months (95% CI: 16.75–39.29) and left-sided MCO was 18.23 mo (95% CI: 14.61–21.85) (*P* = .095). CI = confidence interval, MCO = malignant colorectal obstruction.

Clinical success was achieved in 97.1% (65/67) of patients with right-sided MCO and 88.2% (353/400) of patients with left-sided MCO, which was significantly different (*P* = .026). Complication rates were 10.1% for right-sided MCO and 19.9% for left-sided MCO (*P* = .057). Acute and delayed complication rates were 1.4% and 8.7% in right-sided MCO, respectively, and 7.2% and 13.8% in left-sided MCO, respectively (*P* = .069 for acute complications and *P* = .249 for delayed complications). Stent migration rates were 1.4% and 7.5% in right-sided MCO and left-sided MCO, respectively (*P* = .062). The perforation rates were 0% for right-sided MCO and 5% for left-sided MCO (*P* = .058). No significant differences were found in stent ingrowth or outgrowth between the 2 groups. Of the patients with right-sided MCO, 30.4% underwent surgery after stent insertion, while 29.3% of patients with left-sided MCO underwent surgery (Table 2). Mean follow-up times for right-sided and left-sided MCO were 15.31 ± 13.80 months and 18.49 ± 20.10 months, respectively. The mean OS was 28.02 months (95% CI: 16.75–39.29) for right-sided MCO and 18.23 months (95% CI: 14.61–21.85) for left-sided MCO (*P* = .095; Fig. 1).

**Table 2:** Clinical outcomes and details of complications after SEMS placement.

Variable	Overall N = 469	Right-sided N = 69 (%)	Left-sided N = 400 (%)	*P* value
Clinical success	420/469 (89.6)	67 (97.1)	353 (88.2)	.026
Complication	86 (18.3)	7 (10.1)	79 (19.9)	.057
Acute complication (before 15 d)	30 (6.4)	1 (1.4)	29 (7.2)	.069
Delayed complication (after 15 d)	61 (13.0)	6 (8.7)	55 (13.8)	.249
Stent migration	31 (6.6)	1 (1.4)	30 (7.5)	.062
Stent ingrowth	27 (5.8)	4 (5.8)	23 (5.8)	.988
Stent outgrowth	9 (1.9)	2 (2.9)	7 (1.8)	.521
Perforation	20 (4.3)	0	20 (5.0)	.058
Bacteremia/fever	4 (0.9)	0	4 (1.0)	.404
Bleeding	0 (0.2)	0	1 (0.3)	.678
Operation after stent	138 (29.4)	21 (30.4)	117 (29.3)	.842

SEMS = self-expandable metal stent.

### 3.3. Factors associated with clinical success in palliative SEMS placement

Table 3 shows the factors associated with the clinical success of palliative SEMS placement. In univariate logistic regression analysis, right-sided tumor location, length of obstruction, and the use of uncovered stents were significant factors for the clinical success of SEMS placement (OR 4.460 [95% CI: 1.058–18.807], *P* = .042; OR 0.960 [95% CI: 0.937–0.985], *P* = .001; and OR 3.295 [95% CI: 1.801–6.027], *P* < .001, respectively). No significant difference was found between clinical success in terms of age, sex, BMI, PS score, abdominal operation history, location of tumor, metastasis, and length of stent in the univariate logistic regression analyses. In multivariate logistic regression analysis, T3 stage tumor and the use of uncovered stents were significant factors for the clinical success of SEMS placement (OR 8.475 [95% CI: 1.353–52.632], *P* = .022; and OR 9.109 [95% CI: 1.463–56.715], *P* = .018, respectively).

**Table 3:** Univariate and multivariate analyses of the predictive factors of clinical success.

	Univariate analysis			Multivariate analysis		
Variable	OR	95% CI	*P* value	OR	95% CI	*P* value
Age	1.000	0.978–1.023	.999			
Gender, male	1.050	0.577–1.910	.873			
BMI (SD), kg/m^2^	1.070	0.967–1.183	.190			
Hypertension	0.962	0.529–1.749	.898			
Diabetes mellitus	2.038	0.841–4.937	.115			
PS score (0–2)	0.761	0.393–1.472	.417			
Abdomen operation history, yes	1.187	0.572–2.465	.646			
Tumor location (right)	4.460	1.058–18.807	.042	1.477	0.125–17.465	.757
Tumor location (flexure)	1.057	0.430–2.602	.903			
Length of obstruction (mm)	0.960	0.937–0.985	.001	0.961	0.906–1.018	.175
T3 versus T4	4.762	0.878–25.641	.070	8.475	1.353–52.632	.022
M stage	1.042	0.353–3.078	.940			
Peritoneal carcinomatosis	1.477	0.771–2.832	.240			
Uncovered stent	3.295	1.801–6.027	.000	9.109	1.463–56.715	.018
Length of stent (mm)	0.992	0.976–1.009	.373			

BMI = body mass index, CI = confidence interval, OR = odds ratio, PS score = performance status score, SD = standard deviation.

### 3.4. Risk factors of complications after palliative SEMS placement

In univariate logistic regression analysis, PS score of 0 to 2 and the use of covered stent were significant independent risk factors for complications (OR 2.179 [95% CI: 1.136–4.179], *P* = .019; and OR 3.126 [95% CI: 1.934–5.053], *P* < .001, respectively). There was no significant difference between complications in terms of age, sex, BMI, abdominal operation history, location of tumor, length of obstruction, T stage, metastasis, and stent length in univariate logistic regression analyses. In multivariate logistic regression analysis, PS score of 0 to 2 and the use of covered stent were significant independent risk factors for complications (OR 2.466 [95% CI: 1.257–4.839], *P* = .009; and OR 3.035 [95% CI: 1.842–5.002], *P* < .001, respectively; Table 4).

**Table 4:** Univariate and multivariate analyses of the risk factors of complication.

	Univariate analysis			Multivariate analysis		
Variable	OR	95% CI	*P* value	OR	95% CI	*P* value
Age	0.988	0.971–1.005	0.179			
Gender, male	0.746	0.466–1.194	0.222			
BMI (SD), kg/m^2^	0.998	0.927–1.076	0.965			
PS score (0–2)	2.179	1.136–4.179	0.019	2.466	1.257–4.839	.009
Abdomen operation history	0.791	0.442–1.413	0.428			
Tumor location (left)	2.180	0.961–4.946	0.062	2.145	0.926–4.970	.075
Tumor location (flexure)	0.761	0.359–1.612	0.476			
Length of obstruction (mm)	1.006	0.985–1.028	0.566			
T4 versus T3	1.753	0.664–4.627	0.257			
Metastasis	1.477	0.558–3.908	0.432			
Peritoneal carcinomatosis	1.052	0.648–1.708	0.837			
Covered stent	3.126	1.934–5.053	0.000	3.035	1.842–5.002	<.001
Length of stent (mm)	1.012	0.999–1.025	0.071	1.009	0.994–1.023	.242

BMI = body mass index, CI = confidence interval, OR = odds ratio, PS score = performance status score, SD = standard deviation.

### 3.5. PS score and details of complications after palliative SEMS placement

The complication rates in patients with PS scores between 0 and 2 and those in patients with PS scores between 3 and 4 were 20.7% and 10.7%, respectively (*P* = .017). The stent migration rate was significantly higher when the PS score was between 0 and 2 compared to when it was between 3 and 4 (8.1% vs 1.8%, respectively, *P* = .019; Table 5).

**Table 5:** PS score and details of complications after SEMS placement.

Variable	Overall N = 469	PS score 0 to 2 N = 357 (%)	PS score 3 to 4 N = 112 (%)	*P* value
Complication	86 (18.3)	74 (20.7)	12 (10.7)	.017
Stent migration	31 (6.6)	29 (8.1)	2 (1.8)	.019
Stent ingrowth	27 (5.8)	21 (5.9)	6 (5.4)	.835
Stent outgrowth	9 (1.9)	8 (2.2)	1 (0.9)	.364
Perforation	20 (4.3)	18 (5.0)	2 (1.8)	.137
Bacteremia/fever	4 (0.9)	3 (0.8)	1 (0.9)	.958
Acute complication (before 15 d)	30 (6.4)	22 (6.2)	8 (7.1)	.711

PS score = performance status score, SEMS = self-expandable metal stent.

## 4. Discussion

Palliative SEMS placement for MCO has been considered safe and effective as a first-line treatment. Four systematic reviews and/or meta-analyses compared colonic stent insertion and emergency surgery for the palliation of MCO. Colonic stent placement was associated with a shorter hospital stay, lower intensive care unit admission rate, and earlier initiation of palliative chemotherapy. In addition, the stoma formation rate was significantly lower in the colonic stent placement group than in the emergency operation group.^[10,11,13,14]^ The latest guideline published in 2020 by European Society of Gastrointestinal Endoscopy (ESGE) strongly recommends colonic stent insertion in MCO for palliative purposes with high-quality evidence.^[7]^

In this study, we first analyzed the clinical outcomes and complications after palliative SEMS placement for MCO in a total of 469 patients. The overall clinical success rate was 89.6%, which was similar to previous studies.^[10,11]^ The clinical success rates were significantly higher in patients with right-sided MCO than in those with left-sided MCO. In addition, although not statistically significant, patients with right-sided MCO showed lower complication rates and longer mean OS compared to patients with left-sided MCO. There are limited data directly comparing SEMS placement in right-sided and left-sided MCOs. In a study comparing SEMS placement in 37 patients with right-sided MCO and 99 patients with left-sided MCO, the clinical success rates were 78% for the former and 91% for the latter, while the complication rates were not significantly different.^[20]^ Another study reported that the location of the MCO had no significant impact on the treatment outcome in a relatively small series of patients.^[21]^

Traditionally, primary resection and anastomosis has been considered the first-line treatment option for right-sided MCO. However, these patients are often elderly and in poor physical condition due to reduced food intake and general weakness before they present with obstructive symptoms, such as abdominal pain, nausea, and vomiting. In addition, several studies reported that emergency operation for MCO was associated with higher mortality and complication rates compared to elective surgery.^[22,23]^ Therefore, in the emergency setting, alternative treatment options for bowel decompression have been studied. According to a recent systematic review by Amelung et al,^[24]^ compared to emergency surgery, stent placement was associated with lower mortality, less major morbidity, and lower risk of anastomotic leakage.

The placement of SEMS in patients with right-sided MCO is technically challenging. First, the right-sided MCO tends to be more severely obstructed. Fecal material is in the liquid form in the right-sided colon, and its lumen is wider. Therefore, obstructive symptoms or radiologic signs of obstruction are seen in complete obstruction. Second, since oral bowel cleansing agents cannot be used, only the distal bowel can be cleaned. Lastly, the most important factor is that the distance from the anus to the obstruction site is longer and insertion of the colonoscope into the underprepared bowel is technically challenging. Due to these factors, reports have shown lower technical success rates of colonic stent placement in the proximal colon.^[20,25]^ Conversely, recent data shows no significant difference between the technical success rates of stent insertion in different locations.^[21,26]^ These results indicate that palliative SEMS placement is a safe and feasible alternative treatment option for not only left-sided MCOs, but also for the right-sided ones, with high clinical success rates and low complication rates.

Next, we investigated the predictive factors of clinical success rates and risk factors for complications in SEMS placement. Clinical success was defined as the relief of clinical obstructive symptoms and signs and radiologic signs of obstruction within 48 h after SEMS placement. In this study, 103 of the 469 patients had radiologic T stage information. The T stage showed a significant association with the clinical success of SEMS placement. According to the American Joint Committee on Cancer colon cancer staging, T3 is defined as tumor invasion through the muscularis propria into peri-colorectal tissues, and T4 is defined as the tumor penetrating to the surface of the visceral peritoneum or directly invading other organs.^[19]^ The clinical success rates of SEMs placement were 96.9% in T3 stage tumors and 86.8% in T4 stage tumors. In multivariate logistic regression analysis, T3 stage was a significant predictive factor of clinical success of SEMS placement compared to T4 stage. Studies have reported the association of advanced stages and peritoneal carcinomatosis with clinical outcomes.^[27,28]^ However, to our knowledge, no previous studies have investigated the association between the T stage and clinical outcomes of stent insertion in MCO. A possible reason for this result is that the deeper infiltration of the tumor may lead to the fixation of the bowel, leading to decreased peristalsis and limitation of stent expansion after placement.

There are 2 types of SEMSs: covered and uncovered. Covered stents have the advantage of lower tumor ingrowth compared to uncovered stents. However, as covered stents have lower grip power, stent migration is more common. A recent systematic review and meta-analysis by Mashar et al compared the outcome of covered and uncovered stent insertion in MCO. It included 1 randomized controlled trial and 9 observational studies. Uncovered stents were associated with lower complication rates, including stent migration and tumor overgrowth. In addition, uncovered stents showed lower stent reinsertion rates and longer duration of stent patency. The technical and clinical success rates were similar.^[29]^ In the present study, the use of an uncovered stent was a significant predictive factor of clinical success. In addition, covered stent placement was a significant risk factor for complications. ESGE weakly recommends the use of uncovered stents in MCOs for palliative purposes with low-quality evidence.^[7]^ The result of the present study may strengthen the ESGE guideline recommendation.

Lastly, in the multivariate regression analysis, PS score of 0 to 2 was a significant risk factor for complications. In this study, the stent migration rate was significantly higher in patients with PS scores between 0 and 2 compared to those with PS scores between 3 and 4 and other complication rates were similar in both groups. Previously, a similar result was reported about factors associated with stent failure in gastric outlet obstruction.^[30]^ Patients with a PS score between 3 and 4 are confined to their beds or chairs for >50% of their waking hours, leading to limited physical activity. These patients may, therefore, have decreased peristalsis, resulting in lower rates of stent migration.^[31,32]^

However, this study had some limitations. First, the data were obtained retrospectively. Therefore, selection bias was inevitable. Although recent studies reported similar success rates in right-sided and left-sided colon obstruction stenting, endoscopists feel technically more difficult in right-sided MCO stenting. And so, most patients with right-sided MCO and hemodynamic instability or showing sign of sepsis underwent surgery rather than stent insertion. This could lead to better outcomes of right-sided MCO stenting compared to left-sided MCO. Second, the technical success rate could not be assessed because the number of patients who underwent colonoscopy to access the obstruction site without success could not be counted. Third, as the palliative treatment options for MCO are SEMS insertion and emergency operation, comparing these 2 elements would have been beneficial. Fourth, number of patients undergone stent insertion in right-sided MCO is relatively small to perform adequate statistical analysis. Also, patients who were stage II or III cancer but did not undergo operation because of old age or comorbidities were enrolled too. Nevertheless, this study enrolled a large number of patients with right-sided MCO and left-sided MCO compared to previous studies and showed promising results regarding SEMS insertion in right-sided MCO.

## 5. Conclusion

Palliative SEMS placement in right-sided MCO showed better clinical success rates than left-sided MCO. T3 stage was a significant predictive factor associated with clinical success compared to the T4 stage. The use of uncovered stents is recommended in terms of higher clinical success rates and lower complication rates.

## Author contributions

Conceptualization: Oh Hyung-Hoon, Joo Young-Eun.

Data curation: Oh Hyung-Hoon, Cho Sung-Bum, Hong Ji-Yun,

Kim Dong-Hyun, Yang Hee-Chan, Kim Sang-Wook, Lee Jun,

Kim Seong-Jung, Han Yeom-Dong, Seo Geom-Seok, Hong

Gun-Young, Kim Ho-Dong, Myung Dae-Seong, Kim Hyun-

Soo, Joo Young Eun.

Formal analysis: Oh Hyung-Hoon, Cho Sung-Bum, Hong

Ji-Yun.

Investigation: Oh Hyung-Hoon, Hong Ji-Yun, Myung

Dae-Seong.

Resources: Hong Ji-Yun, Kim Dong-Hyun, Yang Hee-Chan,

Kim Sang-Wook, Lee Jun, Kim Seong-Jung, Han Yeom-Dong,

Seo Geom-Seok, Hong Gun-Young, Kim Ho-Dong, Myung

Dae-Seong, Kim Hyun-Soo.

Supervision: Joo Young Eun.

Writing – original draft: Oh Hyung-Hoon, Cho Sung-Bum, Joo

Young Eun.

Writing – review & editing: Joo Young Eun.
